# Dissection of Genotype-Dependent Responses Reveals Leaf Proteome Signatures Associated with Maize Thermotolerance During Flowering Under Enclosure-Imposed Heat Stress

**DOI:** 10.3390/proteomes14020023

**Published:** 2026-04-29

**Authors:** Ruixiang Liu, Xiaohang Li, Zixin Zha, Meijing Zhang, Lingjie Kong, Yakun Cui, Wenming Zhao, Qingchang Meng, Youhua Wang, Yanping Chen

**Affiliations:** 1 Maize Research Center, Jiangsu Agricultural Academy of Sciences, Nanjing 210014, China; liuruixiang@jaas.ac.cn (R.L.); meimei_zmj@163.com (M.Z.); lingkj@126.com (L.K.); cuiyakun1920@163.com (Y.C.); breeding2005@126.com (W.Z.); qcmeng@jaas.ac.cn (Q.M.); 2 Jiangsu Collaborative Innovation Center for Modern Crop Production, College of Agriculture, Nanjing Agricultural University, Nanjing 210095, China; 2023101047@stu.njau.edu.cn (X.L.); 2024801190@stu.njau.edu.cn (Z.Z.); w_youhua@njau.edu.cn (Y.W.)

**Keywords:** maize, enclosure-imposed humid heat shock, flowering-stage stress, thermotolerance, proteomics, genotype × day, heat shock proteins, benzoxazinoid metabolism, protein homeostasis, DIA mass spectrometry

## Abstract

Background: During maize anthesis, heat stress severely limits productivity—particularly under humid conditions where high humidity suppresses transpirational cooling, forcing tissues to endure direct thermal load. Methods: Using field enclosures to impose enclosure-imposed humid heat shock (EHS), we screened 135 maize inbred lines for flowering-stage yield resilience, using grain weight per ear at maturity under EHS relative to the corresponding control (CK) condition as the primary selection criterion. Based on this screen, we selected two tolerant (R025, R100) and two sensitive (R133, R135) genotypes for data-independent acquisition mass spectrometry (DIA-MS) profiling of the tassel-subtending leaf. Results: At baseline, the selected tolerant lines exhibited a constitutively distinct proteomic state, including lower abundance of light-harvesting complex components and higher abundance or detection frequency of several regulatory proteins, including SRK2E/OST1 and HSF-B2a. Under sustained EHS, the selected sensitive lines showed extensive proteomic disruption, including reduced abundance of photosynthesis-related proteins and oxidative phosphorylation, together with increased abundance of proteins associated with endoplasmic reticulum stress responses and protein turnover. In contrast, the selected tolerant lines displayed a more constrained acclimation response, characterized by relative maintenance of photosynthesis-related proteins together with selective increases in chaperone systems (HSP90/sHSPs) and benzoxazinoid biosynthesis-related proteins. Several proteins showed switch-like detection patterns between the selected tolerant and sensitive lines, including TMEM97-like and a peptidyl-prolyl isomerase, indicating potentially distinct regulatory states. Conclusions: These findings suggest that tolerant performance under enclosure-imposed heat stress is associated with a pre-conditioned proteomic state and enhanced protein homeostasis (proteostasis) buffering capacity that may help preserve photosynthetic function during flowering-stage stress. The identified proteins should be regarded as candidate markers requiring further functional validation before any application in breeding programs aimed at improving adaptation to increasingly frequent heat-stress events.

## 1. Introduction

Rising global temperatures threaten crop productivity, and maize (*Zea mays* L.) is particularly sensitive to heat stress during flowering, when elevated temperature reduces pollen viability and kernel set [1,2]. However, the physiological impact of heat is determined not only by air temperature but also by atmospheric humidity, expressed as vapor pressure deficit (VPD) [3]. Under high VPD (dry heat), transpirational cooling can effectively reduce leaf temperature. In this context, survival is frequently a function of hydraulic strategy: root depth, stomatal conductance, and vascular transport efficiency [3,4].

In contrast, humid heat (low VPD) presents a fundamentally different thermodynamic challenge. High ambient humidity suppresses the vapor pressure gradient that drives evaporation, effectively decoupling this mechanism [3,5,6]. Consequently leaf temperature equilibrates with or exceeds air temperatures, forcing plant tissues to endure direct thermal loads. Tolerance under these conditions relies less on hydraulic avoidance and more on intrinsic cellular thermotolerance: the capacity of the proteome to maintain structural and functional integrity [4,7,8]. Distinguishing the molecular basis of tolerance to humid versus dry heat is critical, as their dominant failure modes—direct thermal denaturation versus dehydration—likely engage distinct molecular response networks [9].

Although yield loss under heat stress is expressed in reproductive organs, reproductive resilience depends on whole-plant physiological stability and systemic signaling. The tassel-subtending leaf (TSL) provides a useful indicator tissue for systemic stress responses that integrates stress responses relevant to flowering-stage performance [10]. Its proteomic state can capture genotype-specific regulatory set-points and buffering capacities that contribute to yield stability.

The maintenance of the proteome, or proteostasis, represents a substantial energetic investment under stress. High temperatures favor protein misfolding and aggregation [11]. To counter this, plants deploy molecular chaperones (HSPs), but maintaining this defense incurs a high metabolic cost [9]. A central hypothesis guiding this work is that tolerant genotypes employ a parsimonious strategy—maintaining critical functions without incurring excessive energetic cost—rather than simply mounting a stronger stress response.

Data-independent acquisition mass spectrometry (DIA-MS) has been applied to maize responses to abiotic stress [7,8,12], including combined drought and heat [13,14]. However, proteome-level characterization of genotype-specific responses to flowering-stage enclosure-imposed heat stress under contrasting humidity backgrounds remains limited, particularly under field conditions in which the humidity background can alter the balance between direct thermal load and atmospheric drought stress. To address this gap, we used a polyethylene film enclosure system that elevates canopy temperature while retaining humidity, thereby creating a field-relevant enclosure stress environment. In the 2024 season, this system approximated a lower-VPD humid-heat screening condition, whereas in the 2025 season used for comparative proteomic profiling, the enclosure generated heat stress accompanied by moderate atmospheric drought. We then used DIA-MS to profile the tassel-subtending leaf proteome in maize genotypes with contrasting yield resilience. Our objectives were to: (i) identify constitutive proteomic features associated with tolerant performance under enclosure-imposed heat stress across contrasting humidity backgrounds; (ii) characterize proteomic remodeling in contrasting maize genotypes during sustained stress exposure; and (iii) nominate candidate proteins, including intensity-driven DAPs, detection-pattern candidates, and genotype × day interaction features, for future validation as markers associated with tolerant performance.

## 2. Materials and Methods

### 2.1. Plant Materials and Experimental Design

**Figure 1:**
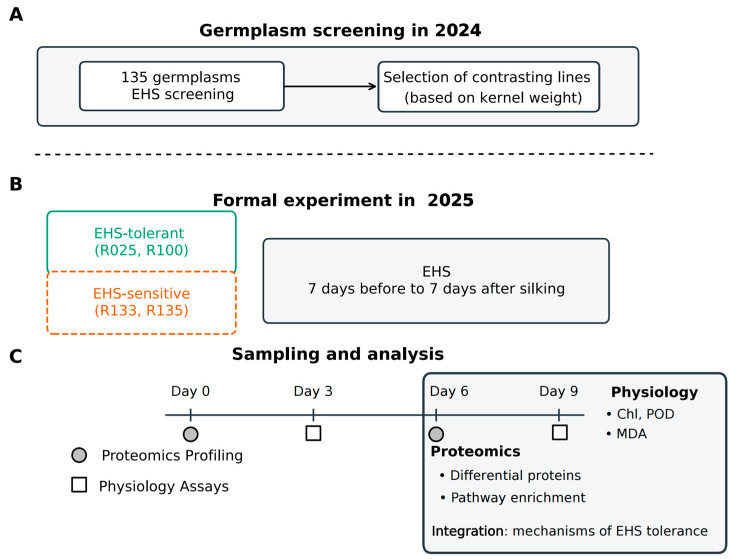
Experimental design and sampling scheme under enclosure-imposed humid heat shock (EHS). (**A**) Germplasm screening (2024): A panel of 135 maize inbred lines was evaluated for flowering-stage EHS tolerance using continuous polyethylene film enclosures. Grain weight per ear at maturity, assessed relative to the corresponding CK condition, was used as the primary criterion for selecting contrasting genotypes. (**B**) Comparative experiment (2025): Two EHS-tolerant lines (R025 and R100; shown in green) and two EHS-sensitive lines (R133 and R135; shown in orange) were grown under CK (open-canopy) and EHS (continuous enclosure) conditions for 21 d spanning anthesis and pollination. (**C**) Sampling and assays: tassel-subtending leaf (TSL) tissue was collected at Days 0, 3, 6, and 9. Physiological traits were measured at Days 3 and 9, and DIA-MS proteomic profiling was performed at Days 0 and 6.

A panel of 135 maize inbred lines (Table S1) was pre-screened in 2024 for flowering-stage tolerance to enclosure-imposed humid heat shock (EHS) using a polyethylene film enclosure system (Figure 1A). The 2024 screening experiment employed a randomized complete block design with two biological replicates (blocks) per treatment: CK (non-enclosed control, open-canopy field condition) and EHS (continuous polyethylene film enclosure treatment). In both treatments, plants of the same lines were grown in the same field background and flowering period.

Tolerance classification in the 2024 screen was based primarily on grain weight per ear measured at maturity, because the purpose of the screen was to identify lines with contrasting yield resilience during flowering-stage stress. Grain weight under EHS was interpreted relative to the corresponding CK performance of the same line. Based on this screen, four lines with contrasting reproductive outcomes were selected for the 2025 comparative experiment: two selected EHS-tolerant lines (R025 and R100; Reid × Tropic heterotic group) and two selected EHS-sensitive lines (R133 and R135, corresponding to the B73 and C72 inbred lines, respectively) (Figure 1B).

For the 2025 experiment, a randomized plot design was used with three biological replicates (blocks) per treatment (CK and EHS). Each plot consisted of a 3 m row with 0.6 m row spacing and 12 plants per row. Enclosures were maintained continuously for 21 d (24 h d^−1^), spanning anthesis and pollination. Canopy temperature and relative humidity were recorded to characterize the treatment microclimate.

### 2.2. Microclimate Monitoring

Canopy air temperature and relative humidity were recorded at 30 min intervals using Elitech GSP-6 data loggers (San Jose, CA, USA). To precisely characterize the stress environment, vapor pressure deficit (VPD), the critical metric for evaporative demand, was calculated using the saturation vapor pressure (SVP) derived from the Arrhenius equation:

VPD = SVP × (1 − RH/100)

$$\mathrm{SVP}=0.61078\;\times \;e^{\frac{17.27*T}{273.5+T}}$$

where T is temperature in °C and RH is relative humidity in percent. Analysis of the 2024 data confirmed that the VPD did not differ significantly between EHS and CK (1.10 vs. 1.08 kPa, *p* > 0.05), confirming the isolation of thermal stress. The 2025 season presented a higher baseline temperature, creating a combined heat and moderate atmospheric drought stress (VPD 2.15 kPa), allowing for the evaluation of genotype stability across varying VPD regimes.

### 2.3. Sampling Strategy for Proteomic and Physiological Assays

The TSL was sampled at four time points: Day 0 (pre-enclosure baseline) and Days 3, 6, and 9 after EHS initiation. Physiological traits (chlorophyll content, peroxidase (POD) activity, and malondialdehyde (MDA) concentration) were measured on Days 3 and 9. Proteomics was performed on Days 0 and 6 (Figure 1C). All sampling was conducted at a fixed time of day, and enclosures were briefly opened to collect tissue. By Day 6 under EHS, the selected sensitive lines showed visible leaf chlorosis and mild scorching, whereas the selected tolerant lines and CK plants remained visually much less affected (Figure S2). In addition to this foliar injury, the selected sensitive lines later exhibited near-complete or complete kernel abortion under EHS, highlighting the severity of their reproductive failure under flowering-stage stress. The temporal design was chosen to capture different layers of the stress response. Days 3 and 9 were used for physiological measurements to represent early and later physiological outcomes, whereas Day 6 was selected for proteomic profiling because it marked an intermediate stage at which the selected sensitive lines had already begun to exhibit visible injury while the selected tolerant lines remained comparatively intact. This timing was considered suitable for capturing biologically informative proteome remodeling before late-stage deterioration became dominant in the selected sensitive lines.

### 2.4. Physiological Measurements

Total chlorophyll was quantified using the Lichtenthaler method [15]. Briefly, fresh leaf tissue was extracted in 80% (*v*/*v*) acetone in the dark, and the absorbance of the clarified extract was measured spectrophotometrically at 663, 645, and 470 nm. Chlorophyll concentration was calculated using standard equations. POD activity was determined by the guaiacol oxidation method using crude enzyme extracts prepared from fresh leaf tissue, and the increase in absorbance at 470 nm was used to calculate enzyme activity. MDA concentration was measured using the thiobarbituric acid (TBA) method. Fresh tissue was homogenized in trichloroacetic acid, reacted with TBA reagent, heated, rapidly cooled, and then centrifuged prior to absorbance measurement at 532 and 600 nm, with correction for nonspecific turbidity as appropriate. Three biological replicates per genotype were analyzed. Statistical significance for physiological traits was assessed using two-way ANOVA with genotype class and sampling day as fixed factors, followed by Tukey’s HSD test where appropriate.

### 2.5. Protein Extraction and DIA-MS Proteomic Analysis

Frozen tassel-subtending leaf (TSL) tissue was ground to a fine powder under liquid nitrogen and lysed for protein extraction. Protein concentration was determined using a bicinchoninic acid (BCA) assay, and protein quality was further assessed by SDS-PAGE to evaluate band integrity. Appropriate aliquots of each protein extract were subjected to denaturation, reduction, and alkylation, followed by tryptic digestion at 37 °C for 2 h. After digestion, the reaction was terminated and the peptide-containing supernatant was desalted using a C18 column. Desalted peptides were concentrated at 45 °C, reconstituted in loading solvent, and used for LC-MS/MS analysis.

Peptide separation was performed on a Vanquish Neo UHPLC system (Thermo Scientific, Waltham, MA, USA). Mobile phase A consisted of 0.1% (*v*/*v*) formic acid in water, and mobile phase B consisted of 0.1% (*v*/*v*) formic acid in 80% (*v*/*v*) acetonitrile. The system was equilibrated with 96% mobile phase A before sample loading. Peptides were first loaded onto a trap column (PepMap Neo, 5 μm C18, 300 μm × 5 mm; Thermo Scientific, Waltham, MA, USA) and subsequently separated on a μPAC Neo High Throughput analytical column (Thermo Scientific, Waltham, MA, USA). The elution gradient was as follows: 0–1.6 min, 4–12% B at 3.0 μL min^−1^; 1.6–2.2 min, 12–14% B at 3.0–1.5 μL min^−1^; 2.2–7.2 min, 14–28% B at 1.5 μL min^−1^; 7.2–9.0 min, 28–45% B at 1.5 μL min^−1^; 9.0–9.2 min, 45–99% B at 1.5–3.0 μL min^−1^; and 9.2–10.0 min, held at 99% B at 3.0 μL min^−1^.

Mass spectrometric analysis was performed on an Orbitrap Astral mass spectrometer (Thermo Scientific, Waltham, MA, USA) operated in positive-ion mode using data-independent acquisition (DIA). The electrospray voltage was set to 1.9 kV. Full MS scans were acquired over an m/z range of 380–980 at a resolution of 240,000, with an automatic gain control (AGC) target of 500% and a maximum injection time of 3 ms. MS/MS scans were acquired at a resolution of 80,000 with an AGC target of 500%, a maximum injection time of 3 ms, and an RF lens setting of 40%. Fragmentation was performed using higher-energy collisional dissociation (HCD) with a normalized collision energy of 25%. The DIA isolation window was set to 2 Th, and the acquisition cycle time was 0.6 s.

The DIA raw files were processed using DIA-NN (v2.2) [16]. Protein identification was performed against the *Zea mays* reference proteome from UniProt (https://www.uniprot.org/proteomes/UP000007305, accessed on 4 August 2025) database using a workflow that combined predicted spectral information with empirically supported spectral evidence to improve identification depth and confidence [17]. Trypsin was specified as the digestion enzyme, with up to two missed cleavages allowed. Quantitative information was extracted from the DIA data using the predicted spectral library together with library information supported by the match-between-runs strategy. Final identifications were filtered at 1% false discovery rate (FDR) at both the precursor and protein levels, and the resulting protein quantification matrix was used for downstream statistical analyses.

### 2.6. Proteomic Data Processing and Statistical Analysis

Protein intensity values exported from DIA-NN were log_2_-transformed and normalized prior to downstream analysis. Data completeness was evaluated by comparing missing-value proportions across samples and experimental groups. Proteins with >75% missing values were removed, and the remaining missing values were imputed using a minimal-value strategy. Replicate reproducibility was assessed by pairwise Pearson correlation analysis and principal component analysis (PCA). The final dataset contained 11,116 protein groups.

Because time-matched Day-6 non-enclosed control samples were not included in the proteomic experiment, the Day 6 versus Day 0 contrasts capture the combined effects of sustained enclosure exposure and temporal/developmental progression. Accordingly, these comparisons are described as proteomic changes under continuous enclosure exposure relative to baseline and are interpreted cautiously. We explicitly acknowledge that, due to the absence of time-matched Day 6 non-enclosed controls, these contrasts reflect a combination of enclosure exposure and developmental progression, which represents an inherent limitation of the experimental design.

Differentially abundant proteins (DAPs; Day 6 versus Day 0) were identified within each tolerance class using the limma package in R with empirical Bayes moderation [18]. Unless otherwise indicated, significance was defined as an adjusted *p*-value < 0.05 after Benjamini–Hochberg correction and an absolute log_2_ fold change (|log_2_FC|) ≥ 1. Genotype × day interaction effects were assessed within the same limma framework, and proteins with interaction FDR < 0.05 were designated candidate interaction proteins.

To capture both quantitative and qualitative proteomic differences, proteins were classified as intensity-driven or detection-driven DAPs. A protein was designated as an intensity-driven DAP only if it was reliably quantified in both conditions under comparison, defined as detection in ≥2/3 replicates per genotype or ≥4/6 replicates in pooled-group analyses. Proteins failing this reproducibility threshold but showing clear group-wise differences in detection frequency were classified as detection-driven candidate proteins. Functional enrichment analysis of Gene Ontology (GO) terms and Kyoto Encyclopedia of Genes and Genomes (KEGG) pathways was performed on the set of intensity-driven DAPs using clusterProfiler [19]. The quantified protein set in the present dataset was used as the enrichment background, and adjusted *p*-values (*q*-values) < 0.05 were considered significant.

### 2.7. Data Availability

Raw mass spectrometry proteomics data have been deposited in the iProX repository [20] (www.iprox.cn, accessed on 22 April 2026) under dataset identifier IPX0015287000. Processed protein intensity matrices and relevant analysis scripts are available from the corresponding author upon request.

### 2.8. Statistical Analysis

Physiological data were analyzed using two-way ANOVA with genotype class and sampling day as fixed effects. Post hoc comparisons were performed using Tukey’s honestly significant difference (HSD) test where appropriate. For proteomic analyses, protein intensities were log2-transformed and normalized prior to statistical testing. Differential abundance analysis was performed using limma with empirical Bayes moderation. Benjamini–Hochberg correction was used to control the false discovery rate in both differential abundance and enrichment analyses. In this manuscript, the terms “adjusted *p*-value” and “*q*-value” refer to multiple-testing-corrected significance values derived from this FDR procedure.

## 3. Results

### 3.1. Field Performance Under Flowering-Stage EHS Identifies Contrasting Response Classes for Downstream Proteomic Comparison

**Figure 2:**
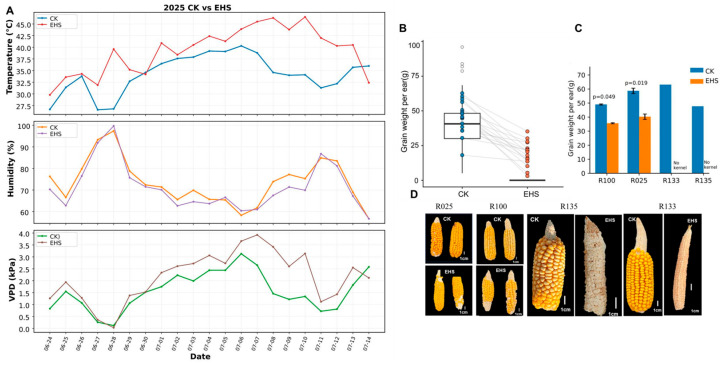
Field microclimate and yield divergence under flowering-stage EHS. (**A**) Time series of canopy temperature, relative humidity, and vapor pressure deficit (VPD) during the daily key stress window (10:00–16:00 h) in CK and EHS plots in the 2025 season. (**B**) Paired comparison of grain weight per ear for the 135-line panel under CK versus EHS. (**C**) Grain weight per ear of selected tolerant and sensitive lines under CK and EHS (mean ± SD, *n* = 3). (**D**) Representative ears from CK and EHS treatments; scale bars = 1 cm.

Across the two growing seasons (2024 and 2025), the polyethylene film enclosure effectively elevated canopy temperature but created distinct microclimatic stress regimes due to contrasting inter-annual climatic backgrounds. During the key stress window (10:00–16:00 h) in 2024, a season characterized by higher ambient humidity, the film enclosure (EHS) increased mean temperature by 4.0 °C compared to control (CK). However, with relative humidity consistently >76% in both treatments, vapor pressure deficit (VPD) did not differ significantly between EHS and CK (1.10 vs. 1.08 kPa, *p* > 0.05) (Figure S1). This resulted in a “pure heat stress” screening environment in 2024, which facilitated initial identification of genotypes sensitive to high temperature per se, independent of strong atmospheric drought. In contrast, the 2025 season was marked by higher baseline temperature and lower humidity. During the same window, EHS increased mean temperature by 4.9 °C relative to CK and significantly elevated mean VPD from 1.57 kPa in CK to 2.15 kPa under film enclosure (*p* < 0.01) (Figure 2A). Consequently, the 2025 environment provided a more severe “combined heat and moderate atmospheric drought” stress. This inter-annual variation offered a unique opportunity to evaluate genotypic stability across varying VPD regimes.

Field screening under flowering-stage EHS revealed substantial variation among 135 maize inbred lines in yield resilience. Under CK conditions, grain weight per ear was broadly maintained across the panel, whereas EHS reduced kernel set and decreased grain weight per ear in most lines (Figure 2B). Based on ear yield retention under EHS relative to the corresponding CK condition, R025 and R100 were classified as tolerant, whereas R133 and R135 were classified as sensitive because they exhibited near-complete or complete kernel abortion under EHS (Figure 2C,D). Under EHS, the selected sensitive lines developed visible leaf chlorosis and apical scorching by Day 6 and subsequently showed severe reproductive failure, whereas the selected tolerant lines showed comparatively limited visible injury and retained substantial grain set (Figure 2C,D and Figure S2).

### 3.2. Global Characterization of the TSL Proteome

**Figure 3:**
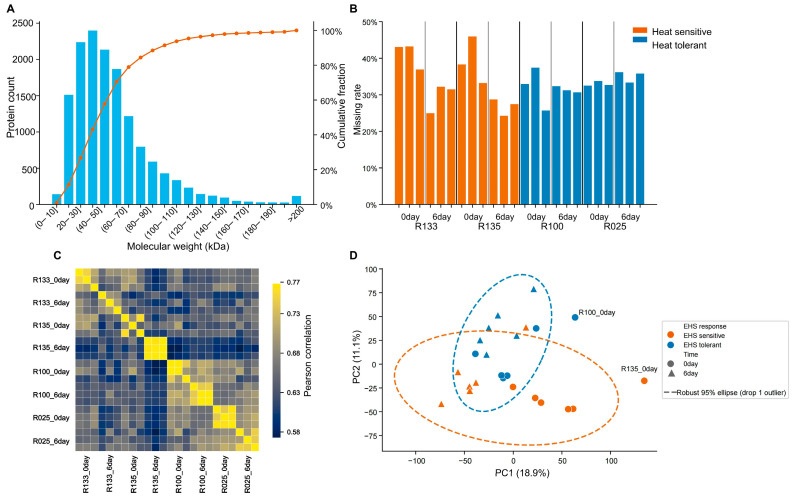
Global quality assessment and multivariate structure of the TSL proteome. (**A**) Distribution of quantified protein groups across molecular weight ranges. (**B**) Missing-value rates across all analyzed samples, showing comparable data completeness among groups. (**C**) Pearson correlation heatmap across biological replicates, indicating high within-group reproducibility. (**D**) Principal component analysis (PCA) of normalized protein intensities showing separation primarily by sampling time and secondarily by tolerance class. Proteomic data were obtained from three biological replicates per genotype per time point (total *n* = 24 samples).

DIA-MS profiling of Day 0 and Day 6 TSL samples quantified 11,116 protein groups (Figure 3A). Data completeness and replicate reproducibility were high, with broadly similar missing-value rates (approximately 30%) across experimental groups and strong within-group correlations (Figure 3B,C). The comparable missing-value distribution across groups suggests that no single condition was disproportionately affected by protein non-detection. Principal component analysis indicated that sampling time was the dominant source of variance in the dataset, separating Day 0 from Day 6 samples along the primary axis, while genotype class also contributed to proteome structure along a secondary axis (Figure 3D).

Across contrasts, a substantial fraction of DAPs displayed group-wise missingness in the raw quantification matrix (Table S2). These proteins were treated as missingness-driven (differential-detection) candidates and summarized primarily by detection frequency, whereas intensity-driven DAPs were prioritized for enrichment analyses and mechanistic interpretation.

### 3.3. Baseline Proteomic Differences Between the Selected Tolerant and Sensitive Lines (Day 0)

**Figure 4:**
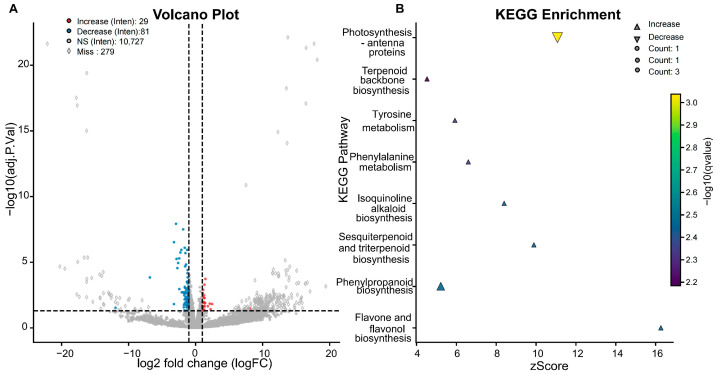
Baseline TSL proteome differences between the selected tolerant and sensitive lines. (**A**) Volcano plot showing differentially abundant proteins (DAPs) in the tolerant versus sensitive comparison at Day 0; significance was defined as |log_2_FC| ≥ 1 and adjusted *p* < 0.05. Open symbols indicate proteins with extensive missingness that were classified as detection-driven candidate proteins rather than intensity-driven DAPs. (**B**) KEGG enrichment analysis of intensity-driven DAPs identified in the tolerant versus sensitive comparison at Day 0. The comparison was based on three biological replicates per genotype at Day 0.

At Day 0, 390 proteins differed in abundance between the selected tolerant and sensitive genotypes (adjusted *p* < 0.05; |log_2_FC| ≥ 1), indicating distinct constitutive proteomic set-points in these contrasting materials (Figure 4A). Among these DAPs, 111 were intensity-driven DAPs, including 29 with increased abundance and 82 with decreased abundance in tolerant relative to sensitive, indicating reliable baseline abundance differences. The remaining 279 DAPs were detection-driven candidate proteins, comprising 232 proteins uniquely or predominantly detected in the tolerant group (with increased detection frequency) and 47 in the sensitive group (with decreased detection frequency) (Figure 4A).

To gain deeper biological insight into pre-existing differences between the selected tolerant and sensitive lines, KEGG analysis was performed on the 111 reliably quantified intensity-driven DAPs. This analysis revealed distinct pathway perturbations in the tolerant genotype at the basal state. The intensity-driven DAPs with increased abundance in the selected tolerant lines were significantly enriched in several metabolic pathways (Figure 4B). Among the significantly enriched pathways, flavone and flavonol biosynthesis and several related specialized-metabolism pathways were represented among proteins with increased abundance in the selected tolerant lines at baseline. By contrast, proteins with decreased abundance in the selected tolerant lines were significantly enriched in photosynthesis—antenna proteins. Because photosynthesis-related proteins are inherently abundant in leaf tissue, this enrichment should not be interpreted on pathway ranking alone. Rather, its biological relevance is supported by the coordinated decrease of multiple light-harvesting components within the quantified dataset. We interpret this pattern as a constitutively distinct state that may reflect a form of pre-conditioning. One possible explanation is that reduced abundance of light-harvesting complex components may help moderate excitation pressure under conditions in which downstream carbon fixation is heat-limited [9]. However, this interpretation remains a working hypothesis and will require further validation across broader genotype sets and functional experiments.

Multiple regulatory or stress-associated proteins, such as HSF-B2a/HSF14 (Zm00001eb009170) and STK1 (Zm00001eb057180), as well as protein quality control-related proteins such as SRK2E/OST1 (Zm00001eb051510) and the deubiquitinase UCHL1 (Zm00001eb134530), were consistently detected in the selected tolerant lines but were not detected, or were rarely detected, in the selected sensitive lines at baseline. These proteins should be regarded as candidate baseline markers associated with contrasting response classes rather than as proven candidate proteins associated with tolerant performance. Additional proteins showing tolerant specific baseline detection included Zm00001eb020560, Zm00001eb033150 (BGGP), and Zm00001eb231840 (MraW family); by Day 6, BGGP and MraW became detectable in the selected sensitive lines (Table S2).

### 3.4. Proteomic Responses Under Sustained Enclosure Exposure (Day 6 Versus Day 0)

To investigate proteomic responses associated with enclosure-imposed heat shock in maize, we conducted a comparative proteomic analysis of selected sensitive and selected tolerant lines subjected to 6 days of treatment. Because time-matched non-enclosed Day 6 samples were not included, the Day 6 versus Day 0 comparison should be interpreted as proteomic remodeling under sustained enclosure exposure relative to baseline, rather than as a strict heat-only effect separated from developmental progression. Differentially abundant proteins were categorized into intensity-driven DAPs (intensity-based differences) and detection-driven candidate proteins (frequency-based detection differences). To improve interpretive clarity, the results below distinguish between intensity-driven and detection-driven protein changes and emphasize cautious interpretation of Day 6 versus Day 0 contrasts in the context of sustained enclosure exposure.

#### 3.4.1. Divergent Proteomic Responses of Selected EHS-Sensitive and Tolerant Lines to Treatment

**Figure 5:**
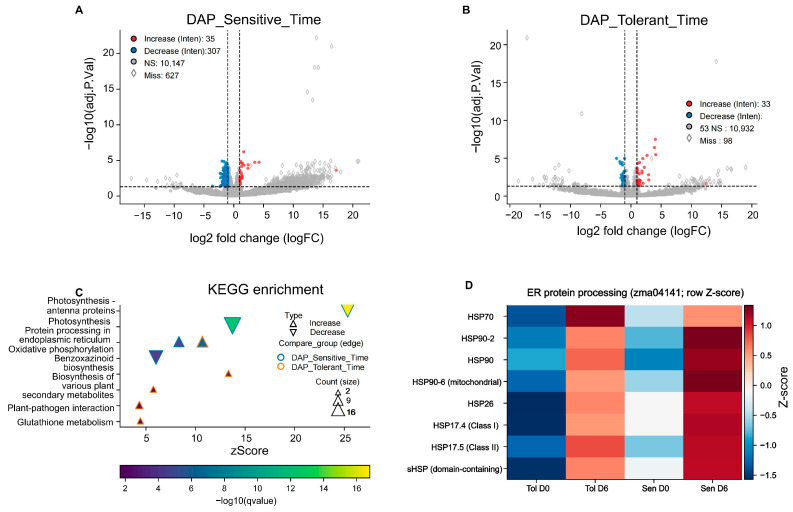
Proteomic responses under sustained enclosure exposure in the selected tolerant and sensitive lines. (**A**,**B**) Volcano plots showing differentially abundant proteins (DAPs) in the Day 6 versus Day 0 comparison for the sensitive and tolerant groups, respectively. Open symbols indicate proteins with extensive missingness that were classified as detection-driven candidate proteins rather than intensity-driven DAPs. (**C**) KEGG enrichment analysis of intensity-driven DAPs showing increased or decreased abundance in each group. (**D**) Heatmap of selected DAPs annotated to the pathway “protein processing in endoplasmic reticulum”. Proteomic comparisons were based on three biological replicates per genotype at each time point.

A total of 969 proteins showed differential abundance in the selected EHS-sensitive lines between Day 6 and Day 0, comprising 342 intensity-driven DAPs (35 with increased abundance and 307 with decreased abundance) and 627 detection-driven candidate proteins, of which 609 were frequently detected on Day 6 but barely detectable on Day 0, and 18 were frequently detected on Day 0 but barely detectable on Day 6 (Figure 5A). The widespread decrease in abundance of many proteins in the sensitive group is consistent with severe stress-associated disruption of cellular functions, potentially involving protein turnover and/or reduced synthesis. In contrast, the tolerant group exhibited a more limited and selective remodeling of specific defense- and stress-related pathways. In the selected EHS-tolerant lines, a total of 184 proteins showed differential abundance between Day 6 and Day 0, comprising 86 intensity-driven DAPs (33 with increased abundance and 53 with decreased abundance) and 98 detection-driven candidate proteins. Among the detection-driven candidate proteins, 52 were frequently detected on Day 6 but barely detectable on Day 0, while 46 showed the opposite pattern (Figure 5B).

#### 3.4.2. Marked Reduction of Energy Metabolism-Related Proteins in Sensitive Maize

KEGG enrichment analysis of intensity-driven DAPs with decreased abundance in the sensitive line revealed a marked disruption of proteins associated with energy production. The DAPs with decreased abundance were strongly and significantly enriched in pathways related to photosynthesis and energy metabolism (Figure 5C). The most highly enriched pathway was “Photosynthesis—antenna proteins” (zma00196), with 12 core proteins showing decreased abundance (fold enrichment = 55.50, *p*.adjust = 1.89 × 10^−17^), followed by decreased abundance of 16 proteins in “Photosynthesis” (zma00195) (fold enrichment = 13.68, *p*.adjust = 4.52 × 10^−13^) and 11 proteins in “Oxidative phosphorylation” (zma00190) (fold enrichment = 5.14, *p*.adjust = 1.14 × 10^−4^) (Table S3). The coordinated decrease in these pathways indicates severe disruption of bioenergetic capacity in the sensitive genotype after six days of heat stress. A reduced energy supply would be expected to constrain ATP-dependent protein quality-control systems, potentially exacerbating stress-associated proteome instability.

#### 3.4.3. Conserved Response: Protein Processing in the Endoplasmic Reticulum

Both genotypes showed increased abundance of proteins in the “Protein processing in endoplasmic reticulum” pathway (zma04141), a common stress response mechanism (Figure 5C). However, enrichment of this pathway appeared more prominent in the tolerant group, with 7 proteins showing increased abundance together with higher fold enrichment (18.23) and stronger adjusted significance (*p*.adjust = 3.76 × 10^−7^), compared with 6 proteins showing increased abundance in the sensitive group (fold enrichment = 13.54) (Figure 5D). This enhanced response is consistent with the possibility that tolerant lines engage a more sustained or better maintained ER-associated protein quality-control response for managing heat-induced protein misfolding.

#### 3.4.4. Increased Abundance of Proteins in Specialized Defense and Antioxidant Pathways in Tolerant Maize

In response to heat stress, the tolerant lines showed increased abundance of proteins in distinct metabolic pathways associated with specialized defense and redox regulation (Figure 5C). Notably, the “Benzoxazinoid biosynthesis” pathway (zma00402) was highly enriched (fold enrichment = 92.45), suggesting that this branch of secondary metabolism may be involved in the tolerant response. This was accompanied by a significant increase in the abundance of proteins in the “Biosynthesis of various plant secondary metabolites” pathway (zma00999). Regarding redox homeostasis, the abundance of proteins in the “Glutathione metabolism” pathway (zma00480) was specifically increased (fold enrichment = 12.33), indicating enhanced capacity for reactive oxygen species scavenging (Table S3). Furthermore, enrichment of the “Plant–pathogen interaction” pathway (zma04626) with 3 proteins showing increased abundance implies a cross-tolerance mechanism, whereby biotic defense signaling may be recruited to mitigate abiotic thermal damage.

### 3.5. Identification of Interaction-Specific DAPs (Genotype × Day)

**Figure 6:**
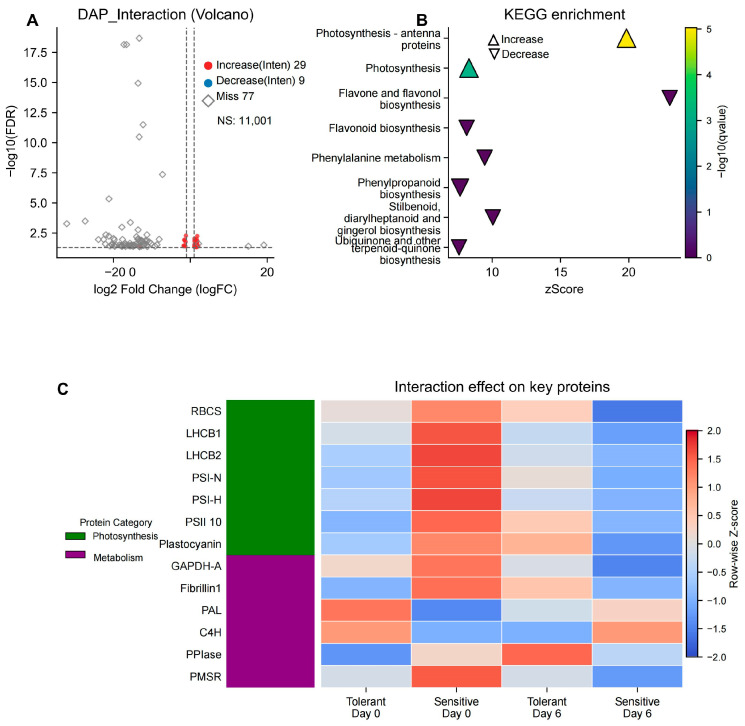
Proteins with significant genotype × day interaction effects under enclosure-imposed heat stress. (**A**) Volcano plot of genotype × day interaction estimates, representing differences in the Day 6 versus Day 0 response between the tolerant and sensitive groups. (**B**) KEGG enrichment analysis of intensity-driven proteins with significant genotype × day interaction effects. (**C**) Heatmap of selected proteins with significant genotype × day interaction effects. Interaction analysis was based on three biological replicates per genotype per time point.

To identify proteins showing response patterns that differed between the selected tolerant and sensitive groups, we used a linear model (limma) to detect differentially abundant proteins with a significant genotype × day interaction effect. These interaction-specific DAPs represent proteins whose response to EHS is statistically distinct between the two EHS-response maize types. A total of 38 proteins were identified under the intensity-driven DAP category (high-confidence quantification with minimal missing values), comprising 29 with increased abundance and 9 with decreased abundance in the interaction contrast, while 77 proteins were classified as detection-driven candidate proteins (Figure 6A).

#### 3.5.1. KEGG Enrichment of Interaction DAPs: Differential Stability of Energy-Related Pathways

To pinpoint biological processes defining superior performance of the tolerant genotype, KEGG enrichment analysis was performed on the intensity-driven DAPs interaction DAPs. The analysis revealed distinct pathway enrichments for the subsets with increased and decreased abundance, highlighting divergent regulatory strategies under heat stress.

The 29 intensity-driven DAPs interaction DAPs with increased abundance were significantly enriched in two energy-related pathways, “Photosynthesis—antenna proteins” (zma00196) and “Photosynthesis” (zma00195) (Table S3) (Figure 6B). The antenna proteins pathway exhibited an exceptional fold enrichment of 132.67 (*p*.adjust = 1.23 × 10^−5^), containing three chlorophyll a-b binding proteins with increased abundance (Zm00001eb433540, Zm00001eb066480, Zm00001eb233170). The photosynthesis pathway also included three proteins (photosystem I reaction center subunit VI, Zm00001eb295610; oxygen-evolving enhancer protein 3-1, Zm00001eb323460; plastocyanin, Zm00001eb379570) showing increased abundance with fold enrichment of 24.53 (*p*.adjust = 0.001). Because these photosynthetic components showed strong decreases in abundance in the sensitive group under heat stress (Section 3.4), their positive interaction effect supports the interpretation that the tolerant group shows greater relative stability of its light-harvesting complex and electron transport chain under prolonged stress.

In contrast, the nine intensity-driven DAPs interaction DAPs with decreased abundance were significantly enriched in pathways related to secondary metabolism, including “Phenylalanine metabolism” (zma00360, fold enrichment = 45.49, *p*.adjust = 0.011), “Phenylpropanoid biosynthesis” (zma00940, fold enrichment = 11.48, *p*.adjust = 0.013), and “Flavone and flavonol biosynthesis” (zma00944, fold enrichment = 132.67, *p*.adjust = 0.033) (Table S3). These pathways involved core enzymes such as phenylalanine ammonia-lyase (PAL, Zm00001eb185250) (Figure 6C). The distinct regulation of the phenylalanine-to-phenylpropanoid transition suggests that the tolerant line may divert carbon flux away from standard secondary growth (e.g., lignin biosynthesis) toward more specific thermal-protective metabolites or antioxidant precursors.

#### 3.5.2. Switch-like Interaction Proteins Based on Detection-Frequency Crossover

Among detection-driven candidate proteins interaction candidates, some proteins showed pronounced mirror-image detection patterns between tolerance classes based on detection frequency across replicates (Table S2), such as TMEM97-like/EXPERA (Zm00001eb305670), HSF-B2a/HSF14 (Zm00001eb009170), dirigent protein (Zm00001eb124210), RABC2a GTPase (Zm00001eb222340), and an uncharacterized protein (Zm00001eb139420). Across these candidates, the selected tolerant lines showed high baseline detection (Day 0) followed by reduced detection at Day 6, whereas the selected sensitive lines showed the inverse pattern. This “mirror-image” dynamic suggests that these proteins may represent candidate regulatory nodes associated with distinct stress-response trajectories in the tolerant and sensitive groups, although their precise functional roles remain to be tested experimentally. Notably, peptidyl-prolyl isomerase (PPIase, Zm00001eb037280) was consistently detected in the sensitive genotype under both control (Day 0) and heat stress (Day 6) conditions (detection frequency: 6/6 at both time points), yet it was completely undetectable in the tolerant genotype at either time point (detection frequency: 0/6). This binary on/off pattern suggests that the selected tolerant lines may rely on a distinct protein-folding environment in which this specific isomerase is not detected. At present, however, whether this protein contributes functionally to stress sensitivity or simply marks a broader proteomic state remains unresolved and will require experimental validation.

### 3.6. Physiological Assays Support the Observed Proteomic Differences

**Figure 7:**
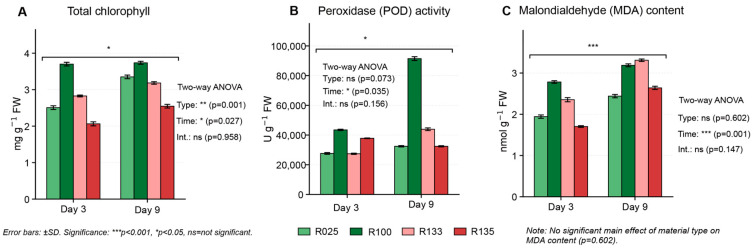
Physiological responses of maize lines under enclosure-imposed heat stress. (**A**) Total chlorophyll content measured at Days 3 and 9. (**B**) Peroxidase (POD) activity measured at Days 3 and 9. (**C**) Malondialdehyde (MDA) content measured at Days 3 and 9. Data are shown as mean ± SD (*n* = 3 biological replicates). Statistical significance was assessed by two-way ANOVA with genotype class and sampling day as fixed factors, followed by Tukey’s HSD test where appropriate.

Physiological measurements aligned with proteomic trends. Total chlorophyll content decreased under stress in all genotypes, but the selected tolerant lines maintained significantly higher chlorophyll at both Day 3 and Day 9 (significant main effects of genotype and time by two-way ANOVA; Figure 7A). POD activity increased primarily with time under EHS (Figure 7B), and MDA concentration also increased with time (Figure 7C). Genotype effects for POD and MDA were less pronounced than for chlorophyll under these conditions.

## 4. Discussion

### 4.1. EHS as a Low-VPD Test Environment for Humid-Heat-Related Tolerance: Partially Decoupling Thermal Load from Dehydration

The polyethylene film enclosure (EHS) system provided a useful field platform for evaluating flowering-stage heat stress under contrasting humidity backgrounds across years, particularly approximating a lower-VPD scenario in the 2024 season. By maintaining high relative humidity (>76%) and low vapor pressure deficit (VPD), the system approximated a low-VPD heat-stress scenario in which evaporative cooling via transpiration was physically constrained. Under these conditions, leaf temperatures likely approached elevated air temperatures, potentially increasing reliance on intrinsic cellular stability rather than avoidance mechanisms. This low-VPD humid-heat environment represents an increasingly relevant stress scenario [3,5]. The severe failure of the selected sensitive lines under these conditions—despite ample soil moisture—suggests that hydraulic avoidance alone may not be sufficient under humid heatwaves. These observations support the view that intrinsic cellular and metabolic tolerance traits deserve increased attention in future breeding efforts [3].

The observed yield divergence—especially the near-complete or complete kernel abortion in the selected sensitive lines—supports the hypothesis that tissue-level proteomic integrity and metabolic robustness are important contributors to reproductive resilience under humid heat. The fact that the selected sensitive lines exhibited terminal leaf chlorosis while the selected tolerant lines maintained higher chlorophyll content corroborates a model where photosynthetic stability, rather than water status management, may be an important determinant of performance under humid heat [6]. By contrast, the 2025 season, with its elevated VPD, created enclosure-imposed heat stress accompanied by moderate atmospheric drought, suggesting that partially distinct tolerance mechanisms may be mobilized under different microclimatic stress scenarios. Together, these inter-annual EHS environments provide an ecologically relevant screening platform that disentangles heat tolerance per se from tolerance to concurrent heat and atmospheric drought.

### 4.2. Divergent Proteome Trajectories: Broad Disruption Versus Constrained Acclimation

A central finding of this study is the strong contrast in the magnitude and direction of proteome remodeling between the selected tolerant and sensitive response classes. Under sustained enclosure exposure (Day 6 versus Day 0), sensitive lines underwent extensive proteomic remodeling (342 intensity-driven DAPs and 627 detection-driven candidate proteins), characterized by broad decreases in abundance of photosynthesis-associated proteins and concomitant increases in the abundance of ER-associated processing factors, autophagy-related proteins, and components linked to proteolysis. This pattern is consistent with severe bioenergetic disruption and broad impairment of cellular maintenance, in which the ATP demands of protein quality control may increasingly compete with a diminishing energy supply [11]. Such a scenario could promote increased protein turnover, impaired photosynthetic maintenance, and activation of catabolic stress responses [8,21,22]. During flowering, such diminished source capacity constrains assimilate supply to reproductive sinks, aligning with the kernel abortion observed under EHS [23].

In contrast, the selected tolerant lines exhibited a highly constrained and targeted response (184 DAPs, 86 intensity-driven DAPs and 98 detection-driven candidate proteins), marked by increased abundance of chaperone systems (including HSP90 and small HSPs) [24], selective increase in abundance of proteins in specialized metabolism (notably benzoxazinoid biosynthesis), and comparatively stable abundances of photosynthesis-related proteins [22,25,26]. This more constrained remodeling is consistent with a strategy centered on proteome buffering and targeted acclimation rather than broad reprogramming [5,21]. The ability of tolerant genotypes to maintain core functions such as photosynthesis while selectively increasing protective systems is consistent with a thermotolerance pattern that may involve functional stability, in which preservation of ATP-generating capacity may help support the energetic cost of chaperone activity.

The absence of time-matched non-enclosed Day 6 controls limits strict partitioning of enclosure effects from developmental progression. Nonetheless, the magnitude of divergence between tolerance classes, the visible injury in the selected sensitive lines by Day 6, and the concordance between chlorophyll retention and photosynthetic protein stability support the interpretation that enclosure exposure amplifies distinct proteome trajectories [5,6]. Time-matched controls will be required to quantify heat-specific and developmental components [5].

### 4.3. Baseline Proteomic Differences and a Putative Reduced-Antenna Hypothesis

The selected tolerant and sensitive lines occupied distinct constitutive proteomic set-points, with 390 DAPs (111 intensity-driven DAPs and 279 detection-driven candidate proteins) identified at baseline (Day 0). Notably, the selected tolerant lines exhibited significantly lower abundance of certain light-harvesting complex (LHC) components and antioxidant proteins (e.g., LHCB1, GST1, SOD1). One possible interpretation is that reduced baseline antenna capacity may moderate excitation pressure under heat stress. One possible rationale is that when carbon fixation is thermally constrained, for example through the heat sensitivity of Rubisco activase, a larger antenna system may increase excitation pressure by capturing photon energy that cannot be efficiently utilized, thereby promoting photooxidative stress [9]. This baseline pattern may reflect a trade-off between maximal photosynthetic capacity under optimal conditions and greater stability under heat stress, although this possibility remains to be tested more directly.

This interpretation is further supported by the constitutive presence of regulatory and proteostasis-associated proteins in the selected tolerant lines, including SRK2E/OST1 and the deubiquitinase UCHL1. These data are consistent with a proteomic state that may be better prepared for rapid signaling and protein quality-control responses prior to stress onset [21,22,25], although causality cannot be established from the present dataset alone.

### 4.4. Switch-like Interaction Candidates and a Putative ER-Linked Regulatory Hypothesis

Beyond global DAP patterns, a subset of proteins displayed qualitative “mirror-image” detection dynamics between tolerant and sensitive classes. These switch-like candidates may reflect genotype-specific differences in regulatory states associated with transitions from early signaling to later stress responses [5,21,25].

Among the switch-like candidates, the TMEM97-like/EXPERA transmembrane protein (Zm00001eb305670) showed a striking crossover pattern: it was consistently detected at baseline but diminished by Day 6 in tolerant lines, whereas it was absent at baseline and became detectable by Day 6 in sensitive lines. In mammalian systems, TMEM97 has been linked to ER-associated functions and stress-related signaling, but the functions of plant TMEM97-like proteins remain unclear [21]. Thus, an ER-linked signaling or proteostasis-related role in maize remains a plausible but still speculative working hypothesis. The contrasting detection pattern observed here suggests that TMEM97-like may mark distinct stress-response states in the tolerant and sensitive groups, but targeted genetic and cell-biological experiments will be required to clarify its function.

Functional context for additional switch-like proteins. The switch-like trajectory of HSF-B2a/HSF14 suggests qualitative differences in heat-response regulatory circuitry, potentially affecting the timing and amplitude of downstream responses [4,27]. The dirigent protein implicates phenolic and cell-wall-associated processes that may influence structural stability or redox coupling. RABC2a (a vesicle trafficking GTPase) is consistent with differential engagement of membrane trafficking pathways intersecting with vacuolar sorting and autophagy [21,22,25].

Notably, peptidyl-prolyl isomerase (PPIase, Zm00001eb037280) exhibited an extreme on/off pattern: consistently detected in the sensitive genotype under both control and stress conditions, yet completely undetectable in the tolerant genotype at either time point. This qualitative divergence raises the possibility that this specific PPIase may be associated with a stress-sensitive folding environment or with particular heat-labile client proteins [28]. However, whether it contributes functionally to stress sensitivity or simply marks a broader proteomic state remains to be established experimentally. The consistent absence of this PPIase in the selected tolerant lines, coupled with maintained photosynthetic machinery, further supports the interpretation that tolerance stems from a fundamentally different, and perhaps more energy-efficient, protein-folding environment.

### 4.5. Proteostasis Costs Under Humid Heat and Metabolic Reprogramming

Humid heat imposes a direct burden on proteome stability by accelerating protein misfolding while simultaneously limiting transpirational cooling [5,6,22]. Protein quality control is energetically costly: chaperone cycling consumes ATP, ubiquitination and proteasome activity require ATP, and replacement synthesis consumes energy and reducing power [22,25]. The selected sensitive lines exhibited broad increases in ER-associated processing and turnover factors together with reduced abundance of photosynthesis- and energy metabolism-related proteins, consistent with increasing competition between proteostasis costs and maintenance of energy-producing functions [21,22,25]. The selected tolerant lines, in contrast, displayed a more focused increase in chaperones while relatively maintaining photosynthesis-related protein abundance, consistent with potentially more effective proteome buffering [5,21,22,25]. The qualitative absence of PPIase in the selected tolerant lines is consistent with the possibility of a distinct protein homeostasis context, although this interpretation remains provisional.

A distinctive feature of the selected tolerant lines was the specific enrichment of benzoxazinoid biosynthesis (zma00402, fold enrichment = 92.45) and glutathione metabolism (zma00480, fold enrichment = 12.33) pathways. Although benzoxazinoids are best characterized in biotic defense, their strong increase in abundance under EHS suggests a pivotal role in abiotic thermal adaptation. They may contribute to stress adaptation through antioxidant-related or other protective functions, but the precise biochemical role of this pathway in humid heat tolerance remains to be clarified [11]. The metabolic shunt from general phenylpropanoid synthesis to specific BX synthesis may represent a shift in carbon allocation toward specific metabolic pathways.

### 4.6. Interaction Proteins as Candidate Markers and Future Outlook

Genotype × day interaction proteins were enriched for photosynthesis-related components, with LHCB family proteins showing contrasting stability between the selected tolerant and sensitive lines. The positive interaction effect on photosynthesis-related proteins, including chlorophyll a/b-binding proteins, photosystem I reaction center subunit VI, oxygen-evolving enhancer protein, and plastocyanin, supports the interpretation that the selected tolerant lines better maintained their light-harvesting complex and electron transport chain under prolonged enclosure stress than the selected sensitive lines. These proteins therefore represent candidate features associated with relative stability under stress in the present dataset and warrant further validation across broader germplasm and stress contexts.

Together with the switch-like behavior of candidates such as TMEM97-like, HSF-B2a, and the peptidyl-prolyl isomerase, as well as baseline set-point differences and selective increases in chaperones and specialized metabolism, these interaction proteins define a prioritized set of candidate proteins for future validation. At this stage, these proteins should be considered candidate markers associated with flowering-stage humid heat tolerance rather than validated biomarkers ready for deployment in marker-assisted selection. Future work should test their robustness across broader germplasm panels, contrasting humidity regimes, and targeted functional experiments.

A major limitation of this study is the absence of time-matched non-enclosed Day 6 controls, which prevents strict separation of enclosure–exposure effects from developmental progression. This constraint has been explicitly considered in both the interpretation of results and the framing of conclusions. Comparative experiments explicitly contrasting low-VPD humid heat with high-VPD dry heat will be needed to identify regime-specific signatures and general thermotolerance mechanisms.

### 4.7. Limitations and the Unresolved Complexity of the Proteome

While DIA-MS provides deep coverage of the maize proteome, it is important to acknowledge the inherent complexity of the proteome relative to the identified gene products. Regardless of the experimental and analytical strategies employed, the issue of proteoforms—including post-translational modifications (PTMs), splice variants, and proteolytic products—remains a challenge [29]. In this study, protein identification and quantification were primarily based on canonical open reading frame (ORF) products aggregated from peptide precursors. Consequently, distinct proteoforms arising from phosphorylation, acetylation, or alternative splicing may be subsumed under a single protein identifier, potentially masking specific regulatory states [30]. For example, the “switch-like” behavior of candidates such as HSF-B2a may be driven by specific phosphorylation events rather than total protein abundance alone. Future targeted investigations should integrate targeted top-down proteomic approaches [31], isoform-aware inference algorithms [32], and orthogonal transcriptomic data to resolve these specific proteoforms to fully elucidate the functional mechanisms of humid heat tolerance [29,30,31,33].

Interpretation of GO and KEGG enrichment should also be made with caution because annotation coverage in maize remains incomplete and is biased toward conserved and well-characterized proteins. Consequently, enrichment results summarize the annotated fraction of the responsive proteome and may underrepresent poorly characterized or lineage-specific processes.

## 5. Conclusions

By integrating field-scale enclosure-imposed heat stress with high-depth DIA-MS proteomic profiling of the tassel-subtending leaf, this study identified proteomic patterns associated with maize flowering-stage resilience under enclosure stress across contrasting humidity backgrounds. Our data suggest that tolerance is associated not simply with better growth performance, but with a distinct and potentially pre-conditioned proteomic state. Sensitivity was associated with marked disruption of ATP-generating and photosynthesis-related pathways, whereas tolerance was associated with a more constrained acclimation response, including relative maintenance of photosynthesis-related proteins together with selective increases in chaperone systems (HSP90/sHSPs) and remodeling of specialized metabolism, including benzoxazinoid-associated proteins. The identification of qualitative switch-like proteins, such as TMEM97-like and a peptidyl-prolyl isomerase, highlights candidate regulatory nodes for future functional study. Overall, the proteomic signatures identified here provide a resource for future validation studies aimed at improving maize resilience to increasingly frequent humid heat events.

## Data Availability

Mass spectrometry proteomics data have been deposited in the iProX repository under dataset identifier IPX0015287000. Processed intensity matrices and analysis scripts are available from the corresponding author upon request.

## Supplementary Materials

The following supporting information can be downloaded at: https://www.mdpi.com/article/10.3390/proteomes14020023/s1, Figure S1: Time series of canopy temperature, relative humidity, and vapor pressure deficit (VPD) during the daily key stress window (10:00–16:00 h) in CK and enclosure-imposed humid heat shock (EHS) plots in the 2024 season. Figure S2: Representative leaf phenotypes at Day 6 under CK and EHS conditions. The selected sensitive lines exhibit early leaf scorching under EHS, whereas the selected tolerant lines and CK plants show minimal visible injury. Table S1: List of 135 maize inbred lines used for EHS tolerance screening. Table S2: Differentially abundant proteins (DAPs) identified using the limma framework across all comparison groups, including both intensity-driven and detection-driven candidates, together with detection frequency information. Table S3: KEGG enrichment analysis results for intensity-driven DAPs across all comparison groups, including pathway annotation, fold enrichment, and adjusted *p*-values (Benjamini–Hochberg correction).

## Abbreviations

The following abbreviations are used in this manuscript:

CK	control (non-enclosed field condition)
EHS	enclosure-imposed humid heat shock
TSL	tassel-subtending leaf
DIA-MS	data-independent acquisition mass spectrometry
DDA	data-dependent acquisition
DAP	differentially abundant protein
HSP	heat shock protein
HSF	heat shock factor
POD	peroxidase
MDA	malondialdehyde
KEGG	Kyoto Encyclopedia of Genes and Genomes
GO	Gene Ontology
VPD	vapor pressure deficit
ER	endoplasmic reticulum
ERAD	endoplasmic reticulum-associated degradation
PCA	principal component analysis
FDR	false discovery rate
UPR	unfolded protein response
LHCB	light-harvesting chlorophyll a/b-binding protein
PAL	phenylalanine ammonia-lyase
PPIase	peptidyl-prolyl isomerase
MAS	marker-assisted selection
ROS	reactive oxygen species
SVP	saturation vapor pressure
