# Supplementary figures and images for: Dissection of Genotype-Dependent Responses Reveals Leaf Proteome Signatures Associated with Maize Thermotolerance During Flowering Under Enclosure-Imposed Heat Stress
Source: Proteomes. 2026 Apr 29;14(2):23. doi: 10.3390/proteomes14020023 (PMC13214725; doi:10.3390/proteomes14020023)

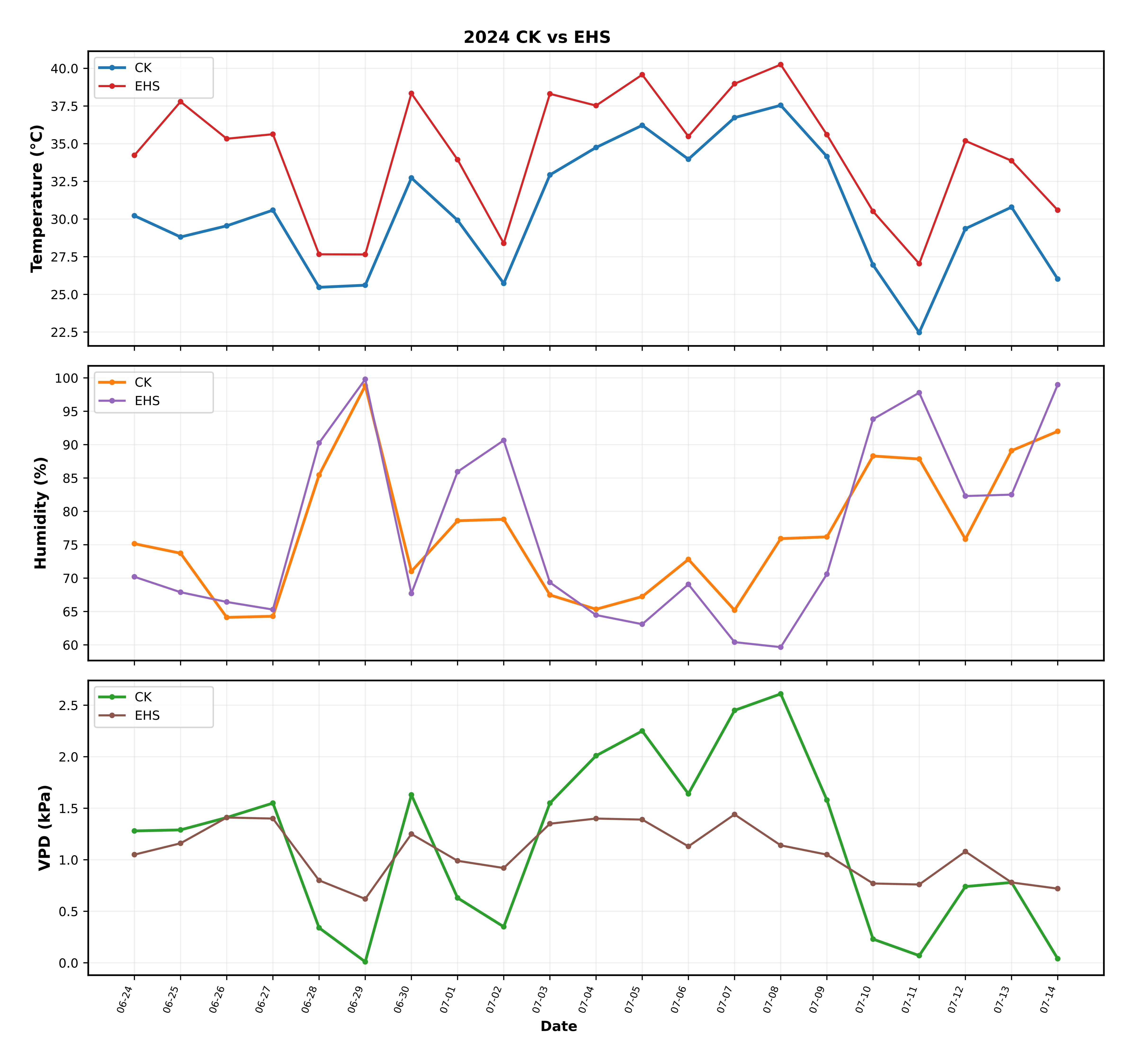

Supplement: Supplementary file 1 [file proteomes-14-00023-s001.zip › Figure S1.png]

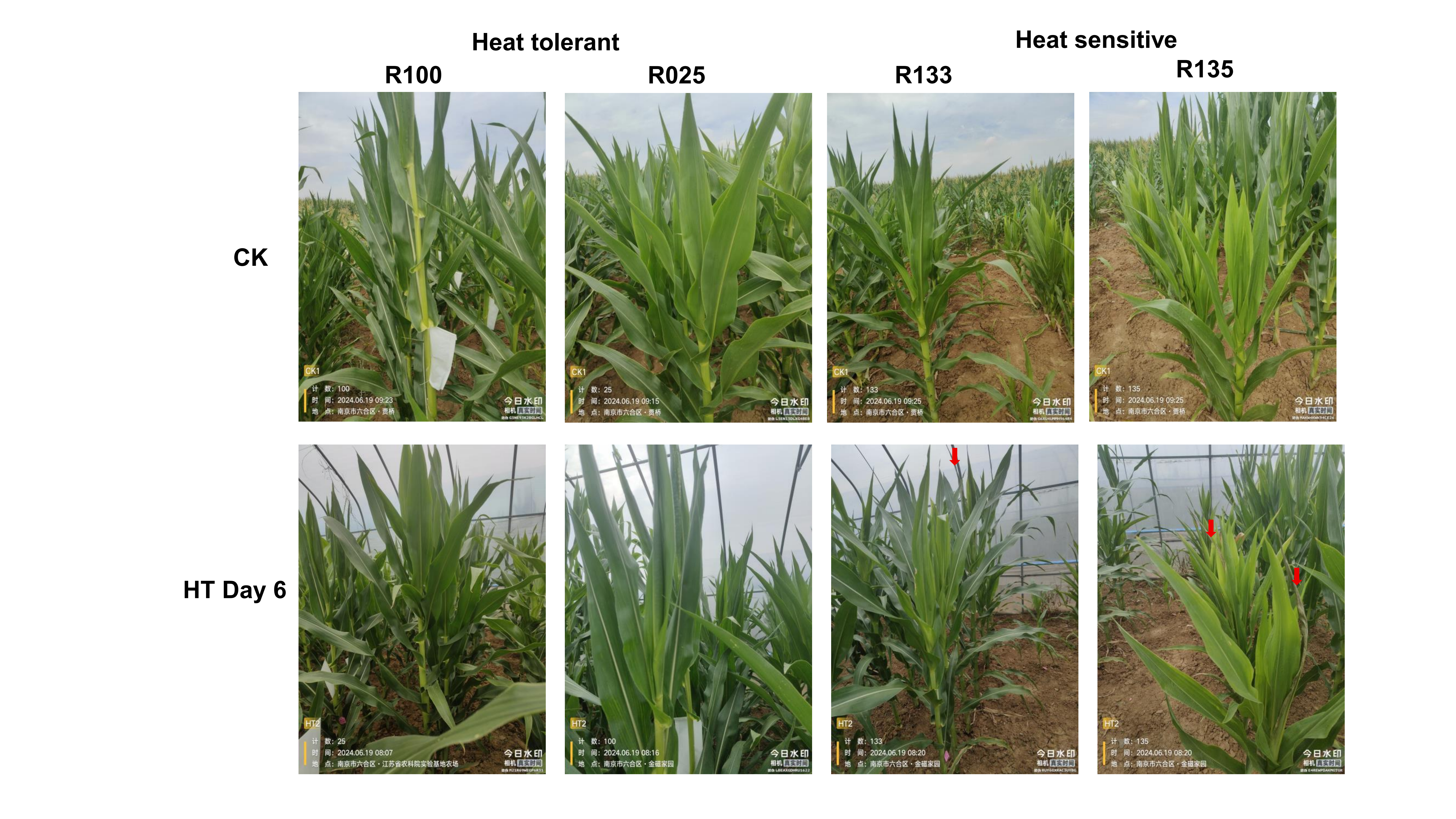

Supplement: Supplementary file 1 [file proteomes-14-00023-s001.zip › Figure S2.png]
